# Cuproptosis-related gene SERPINE1 is a prognostic biomarker and correlated with immune infiltrates in gastric cancer

**DOI:** 10.1007/s00432-023-04900-1

**Published:** 2023-06-15

**Authors:** Leiran Feng, Guixin Li, Dongbin Li, Guoqiang Duan, Jin Liu

**Affiliations:** Department of Gastrointestinal Surgery, The Second Hospital of Hebei Medical University, Shijiazhuang, 050000 China

**Keywords:** GC, SERPINE1, Cuproptosis, Immune, Prognosis

## Abstract

**Purpose:**

The serine protease inhibitor clade E member 1 (SERPINE1) has been studied as a potential biomarker in a variety of cancers, but poorly studied in gastric cancer (GC). The purpose of this study was to explore the prognostic value of SERPINE1 in GC and primarily analyze its functions.

**Methods:**

We analyzed the the prognostic value of SERPINE1 and studied the relationship with clinicopathologic biomarkers in gastric cancer. The expression of SERPINE1 was analyzed by GEO and TCGA databases. Moreover, we validated the results by immunohistochemistry. Next, the correlation analysis between SERPINE1 and the cuproptosis-related genes was analyzed by the “Spearman” method. CIBERSORT and TIMER algorithms were used to analyze the correlation of SERPINE1 with immune infiltration. Furthermore, GO and KEGG gene enrichment analyses were used to study the functions and pathways that SERPINE1 might be involved in. Then, drug sensitivity analysis was performed using CellMiner database. Finally, a cuproptosis-immune-related prognostic model was constructed using genes related to immune and cuproptosis, and verified against external datasets.

**Results:**

SERPINE1 was up-regulated in gastric cancer tissues, which tends toward poor prognosis. Using immunohistochemistry experiment, the expression and prognostic value of SERPINE1 were verified. Then, we found that SERPINE1 was negatively correlated with cuproptosis-related genes FDX1, LIAS, LIPT1, and PDHA1. On the contrary, SERPINE1 was positively correlated with APOE. This indicates the effect of SERPINE1 on the cuproptosis process. Furthermore, by conducting immune-related analyses, it was revealed that SERPINE1 may promote the inhibitory immune microenvironment. The infiltration level of resting NK cells, neutrophils, activated mast cells, and macrophages M2 was positively correlated with SERPINE1. However, B cell memory and plasma cells were negatively correlated with SERPINE1. Functional analysis showed that SERPINE1 was closely related to angiogenesis, apoptosis, and ECM degradation. The KEGG pathway analysis showed that SERPINE1 may be associated with P53, Pi3k/Akt, TGF-β, and other signaling pathways. Drug sensitivity analysis showed that SERPINE1 could be also seen as a potential treatment target. The risk model based on SERPINE1 co-expression genes could better predict the survival of GC patients than SERPINE1 alone. We also verified the prognostic value of the risk score by GEO external datasets.

**Conclusion:**

SERPINE1 is highly expressed in gastric cancer and related to poor prognosis. SERPINE1 may regulate cuproptosis and the immune microenvironment by a series of pathways. Therefore, SERPINE1 as a prognostic biomarker and potential therapeutic target deserves further study.

**Supplementary Information:**

The online version contains supplementary material available at 10.1007/s00432-023-04900-1.

## Background

Gastric cancer (GC) is a common malignant tumor of the gastrointestinal tract, which seriously threatens human health. GC was also reported to be the fifth most common cause of new cases among all cancers and the fourth most common cause of cancer-related death in 2020 (Sung et al. 2021). Thus, it is very important to explore new GC biomarkers and therapeutic targets from the molecular level. Copper is an indispensable cofactor for the life-sustaining activities of all organisms, as it plays an important role in biological processes such as mitochondrial respiration, antioxidant/detoxification, and iron uptake (Ruiz et al. 2021). Cuproptosis is a novel type of copper ion-dependent cell death regulated in cells, which is quite different from the common cell death modes such as apoptosis, pyroptosis, necrotic apoptosis, and ferroptosis. Interestingly, it has recently been reported that cuproptosis-related genes regulate the occurrence and progression of various tumors. Multiple associations have been observed between copper and cancer. Copper is closely related to tumor cell development, angiogenesis, and metastasis (Oliveri 2022). However, the association between cuproptosis and clinical prognosis, tumor microenvironment, and immunotherapy in GC remains unclear. Therefore, we investigated how the cuproptosis-immune-related gene SERPINE1 affects the prognosis of gastric cancer. We may also discover a novel prognostic biomarker and a potential molecular mechanism affecting the prognosis of GC.

The serine family E member 1 (SERPINE1) is a member of the serine protease inhibitor family. It is the primary inhibitor of tissue plasminogen activator (tPA) and urokinase-plasminogen activator (uPA) (Dellas and Loskutoff 2005). Previous literature has shown that SERPINE1 has pro-angiogenesis, growth, migration stimulation, and antiapoptotic activity, all of which are targeted to promote tumor growth, cancer cell survival, and metastasis. SERPINE1 focuses its effect on thrombosis in humans. SERPINE1 is a reliable biological and prognostic marker for a variety of cancers, including breast (Feng et al. 2020), pancreatic (Yu et al. xxxx), bladder, colon (Khoshdel et al. 2016), non-small cell lung (Wang et al. 2021), and low-grade gliomas (Huang et al. 2021). SERPINE1 is involved in immune cell infiltration and plays a role in microenvironmental remodeling and immune cell infiltration in colon cancer (Wang et al. 2021 Jul 3). In recent years, SERPINE1 has been reported to be elevated in gastric adenocarcinoma tissues, and its up-regulation enhances the invasion and proliferation of tumor cells by regulating epithelial–mesenchymal transition (EMT) (Yang et al. 2019).

At present, the establishment of new biomarkers associated with cuproptosis-immune-related pathways is of great importance for the early detection and prognosis of GC. In this study, we conducted a comprehensive analysis of SERPINE1 to investigate the effect of the cuproptosis-immune-related gene SERPINE1 on the tumor microenvironment and survival in GC patients. Immune-infiltration analysis confirmed that SERPINE1 was differentially expressed in tumor tissues. Correlation analysis was conducted between SERPINE1 and other cuproptosis-related genes. Immunoinfiltration analysis was then performed to analyze the role of SERPINE1 in the tumor microenvironment. Gene set enrichment analysis signaling pathway was used to analyze the biological pathways involved in SERPINE1 regulated GC pathogenesis, Drug analysis revealed sensitive drug analysis associated with SERPINE1. Therefore, this study is helpful to show cuproptosis-immune-related genes SERPINE1 as a prognostic marker of GC in the future.

## Materials and methods

### Target gene screening

We downloaded the gastric cancer data (FPKM) from TCGA (https://portal.gdc.cancer.gov/) database, which contains 375 cancer samples and 32 adjacent normal sample. We used samples with complete clinical information for prognostic analysis. GC tissues were compared with normal tissue samples from the TCGA database using the limma package of the R programming language to identify 4656 differentially expressed genes and 501 genes associated with GC prognosis. "Limma" pack sample was used to identify differentially expressed genes (*P* < 0.05 | log2 (FC) |> 1). GSE microarray data were downloaded from the GEO database (http://www.ncbi.nlm.http://nih.gov/geo/). GSE13911 data were obtained. Clinical data of the GSE15459 dataset were obtained for external validation. Next from the Genecard (https://www.genecards.org/) database collecting cuproptosis-related gene 267 (*r* > 7). A total of 4678 immune-related genes were extracted from the innateDB (https://www.innatedb.ca/) database. The target gene SERPINE1 was obtained by intersecting the GC differential expression genes and prognostic-related genes with cuproptosis-related genes and immune-related genes through the Venny diagram.

### Expression validation by immunohistochemistry

Twenty pairs of paraffin-embedded human GC and adjacent normal gastric tissues were collected in the Second Hospital of Hebei Medical University. None of the patients were treated with chemotherapy. Immunohistochemical staining of SERPINE1 (rabbit anti-SERPINE1 polyclonal antibody, Bende Company) was performed at a concentration of 1:100. All sections were evaluated by two physicians and a definitive diagnosis was made. This study was approved by the Ethics Committee of the Second Hospital of Hebei Medical University.

To confirm the expression of SEPINE1 in GC, 20 pairs of matched GC and adjacent gastric tissue samples were selected for immunohistochemical experiments. GC tissue paraffin block routine section was performed. SERPINE1 expression in GC and normal mucosal tissues was detected by streptavidin-peroxidase (SP) assay. Polyclonal antibodies against SERPINE1 were used to evaluate the expression and clinical significance of SERPINE1 in GC. The staining procedure was performed using the SP kit. The presence of strong particle staining in the cell membrane and cytoplasm is considered SERPINE1 positive. Results were determined by visual immune response score (IRS) = staining intensity (IS) multiplied by positive area (AP). IS can be divided into 0 (negative), 1 (weak), 2 (medium), and 3 (strong). The percentage of AP depends on positive cells than 0 (0), 1 (< 1/3), 2 (1/3 to 2/3), 3 (2/3), or higher. IRS 0 ~ 1 is denoted by "-", IRS 2 ~ 3 by " + ", IRS 4 ~ 6 by " +  + ", and IRS6 ~ 9 by " +  +  + ". Thus, SERPINE1 expression is divided into two categories: high (IRS 4–9) and low (IRS 0–3). Each tissue section was independently evaluated by two observers to minimize errors.

### Correlation analysis of cuproptosis-related genes

Cuproptosis-related genes were selected according to previous studies, including FDX1, LIAS, LIPT1, DLD, DLAT, PDHA1, PDHB, MTF1, GLS, and CDKN2A(Wong et al. 2021). The above genes were analyzed by single and multi-gene analysis. The multi-gene correlation map was displayed by the R software package "heatmap". The correlation map of two genes was realized by the R software package "ggstatsplot", and spearman correlation analysis was used to describe the correlation between quantitative variables without normal distribution (*P* < 0.05).

### Immune-infiltration analysis

The “CIBERSORT” algorithm was used to analyze the correlation between SERPINE1 and immune cell infiltration. By TISIDB online database (http://cis.hku.hk/TISIDB/), we validated the correlation between SERPINE1 genes and immune cells. Finally, we also used TIMER2.0 (http://timer.cistrome.org/) database to analyze the correlation of SERPINE1 with immune cell infiltration.

### Enrichment analysis of SERPINE1

GC sample data were downloaded from the CCLE database and co-expression analysis was conducted with target genes. Gene ontological (GO) enrichment analysis and Kyoto Encyclopedia of Genes and Genomes (KEGG) pathway analysis were performed using “clusterProfiler", "ggplot2", "org.Hs.eg.db" packages (*P* < 0.05 & *q value* < 0.2) and verified using the Metascape database (http://metascape.org). To further explore the relationship between the enrichment pathways and the prognosis of GC patients, we performed GSEA analysis to identify SERPINE1-related signaling pathways.

In addition, RNA-sequencing expression profiles and corresponding clinical information for GC were downloaded from the TCGA dataset. R software GSVA package was used to analyze, choosing parameter as method “ssgsea”. The correlation between SERPINE1 genes and pathway scores was analyzed by Spearman correlation.

### Drug sensitivity assessment

We used CellMiner database (www.discover.nci.nih.gov) for drug sensitivity analysis, RNA expression data (RNA: RNA-SEQ) and drug data (Compound activity: DTP NCI-60). The results of drugs approved by Clinical trial and FDA were selected, the Pearson correlation coefficient between SERPINE1 expression and different drugs was calculated. and the analysis results were screened (*P* < 0.05). We analyzed the data with "impute" and "limma" packages.

### Establishment of SERPINE1 co-expression genes-related prognostic risk model

A total of 52 genes related to cuproptosis-immune-related were obtained. We used univariate and multivariate Cox regression analysis to construct a risk model based on the establishment of prognostic risk characteristics related to immunity and cuproptosis. A risk score for each patient was calculated as the sum of each gene’s score, which was obtained by multiplying the expression of each gene and its coefficient. The cohort was divided into high-risk and low-risk groups based on the median value of the risk scores.

Next, survival curves were drawn according to the high- and low-risk groups, and Kaplan–Meier survival curves were used to analyze and compare the survival of the two groups. Draw a risk curve, a risk heat map, etc. To determine whether risk score is an independent prognostic factor for OS in patients with GC, univariate and multivariate independent prognostic analyses were performed combined with a risk score. The accuracy of the prognostic evaluation was shown by the calibration curve, and the survival of patients was predicted by histogram. Combined with the prognostic and clinical features, the ROC curve was constructed to predict the 1-, 3- and 5-year survival rates of GC patients. To make the model more convincing, GEO (GSE15459) validation queues were used to verify the accuracy of the established prognostic features. We used the "glmnet", "timeROC", "survival", and "survminer" packages to build the model and capture the ROC.

### Statistical analysis

All statistical analyses in this paper were carried out by R (version 4.2.1) software and drawing software AI. All statistical analyses were conducted by either the Wilcoxon rank-sum test (two-sided), Pearson’s correlation coefficient test, or Fisher’s exact test (two-sided) as relevant. *P* < 0.05 was considered statistically significant.

## Results

### Cuproptosis-immune-related gene SERPINE1 is highly expressed in gastric cancer and is related to poor prognosis

**Fig. 1:**
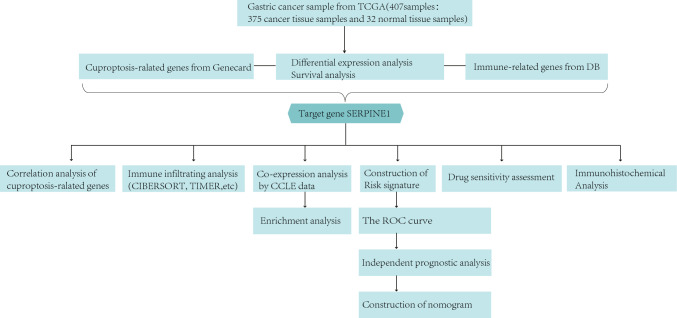
The flowchart of data collection and method implementation

**Fig. 2:**
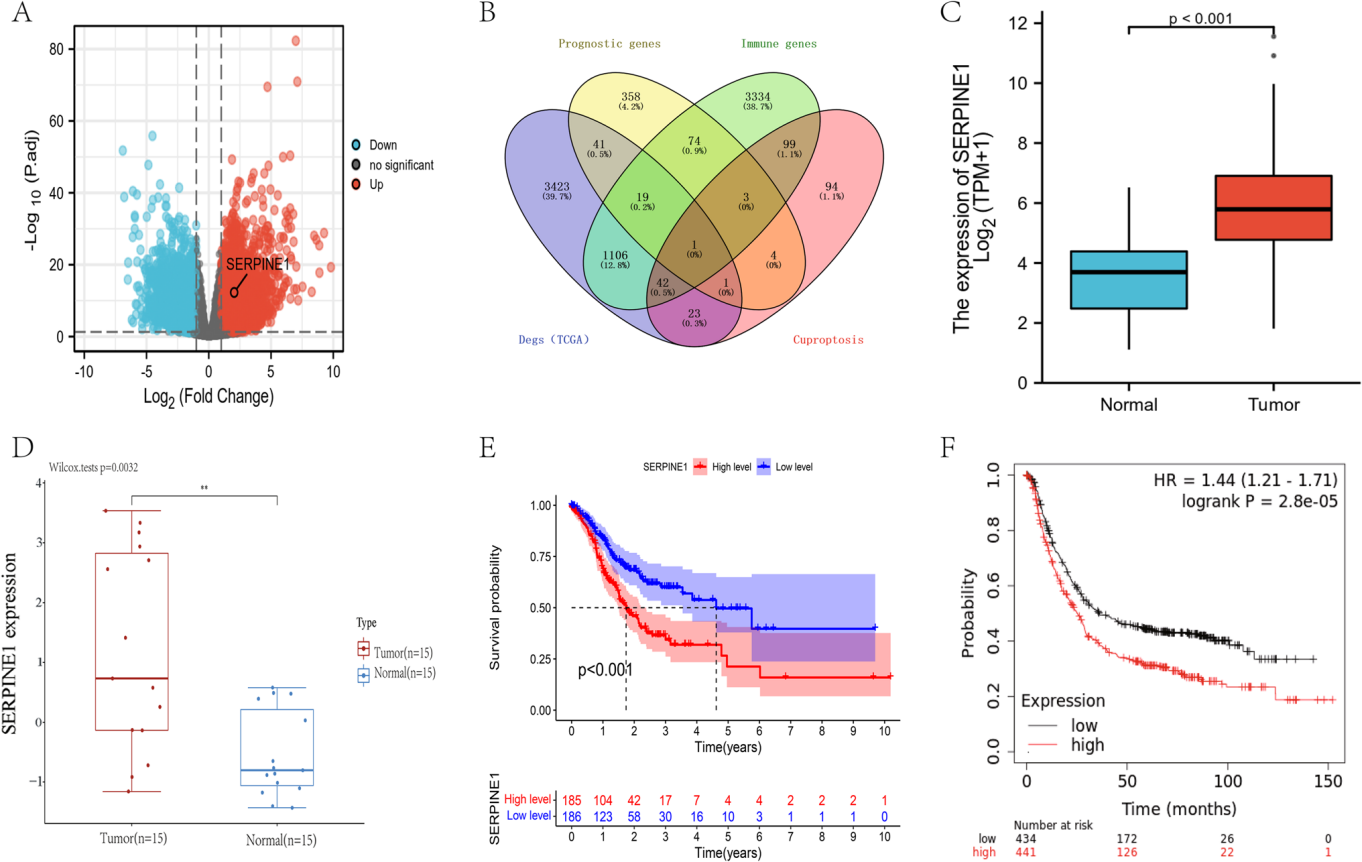
Data collection and analysis. **A** Volcanic map of differentially expressed genes in STAD (TCGA). **B** Venn diagram distribution of differentially expressed genes from STAD; prognostic genes of STAD; cuproptosis-related genes and immune-related genes. **C** Box plot of differentially expressed genes in STAD (TCGA). **D** Box plot of differentially expressed genes in STAD (GSE13911). **E** K–M survival curve in STAD (TCGA). **F** K–M survival curve in STAD (GSE13911). TCGA, The Cancer Genome Atlas; DEGs, differentially expressed genes

To find cuproptosis-related genes related to the prognosis of gastric cancer and further investigate its role in immune infiltration, we conducted this study. A flowchart of the study design is shown in Fig. 1. Volcano maps were used to visualize 4656 DGEs changes between gastric and normal tissues in the TCGA database (Fig. 2A). The target gene SERPINE1 was obtained by intersecting differentially expressed genes and prognostic genes of GC with cuproptosis-related genes and immune-related genes through a Venn diagram (Fig. 2B). After that, differential analysis was then performed to verify the differential expression of SERPINE1 in TCGA (Fig. 2C). Similarly, GSE13911 data from the GEO database showed that the target gene SERPINE1 was also differentially expressed in external data validation sets (Fig. 2D). The next survival curve visualization showed that SERPINE1 had a poor prognosis in the up-regulated gastric cancer group in the TCGA (Fig. 2E). Survival curves for GSE13911 showed that the target gene SERPINE1 also had poor prognostic value in external data validation sets (Fig. 2F).

### Verification the expression of SERPINE1 by IHC

**Fig. 3:**
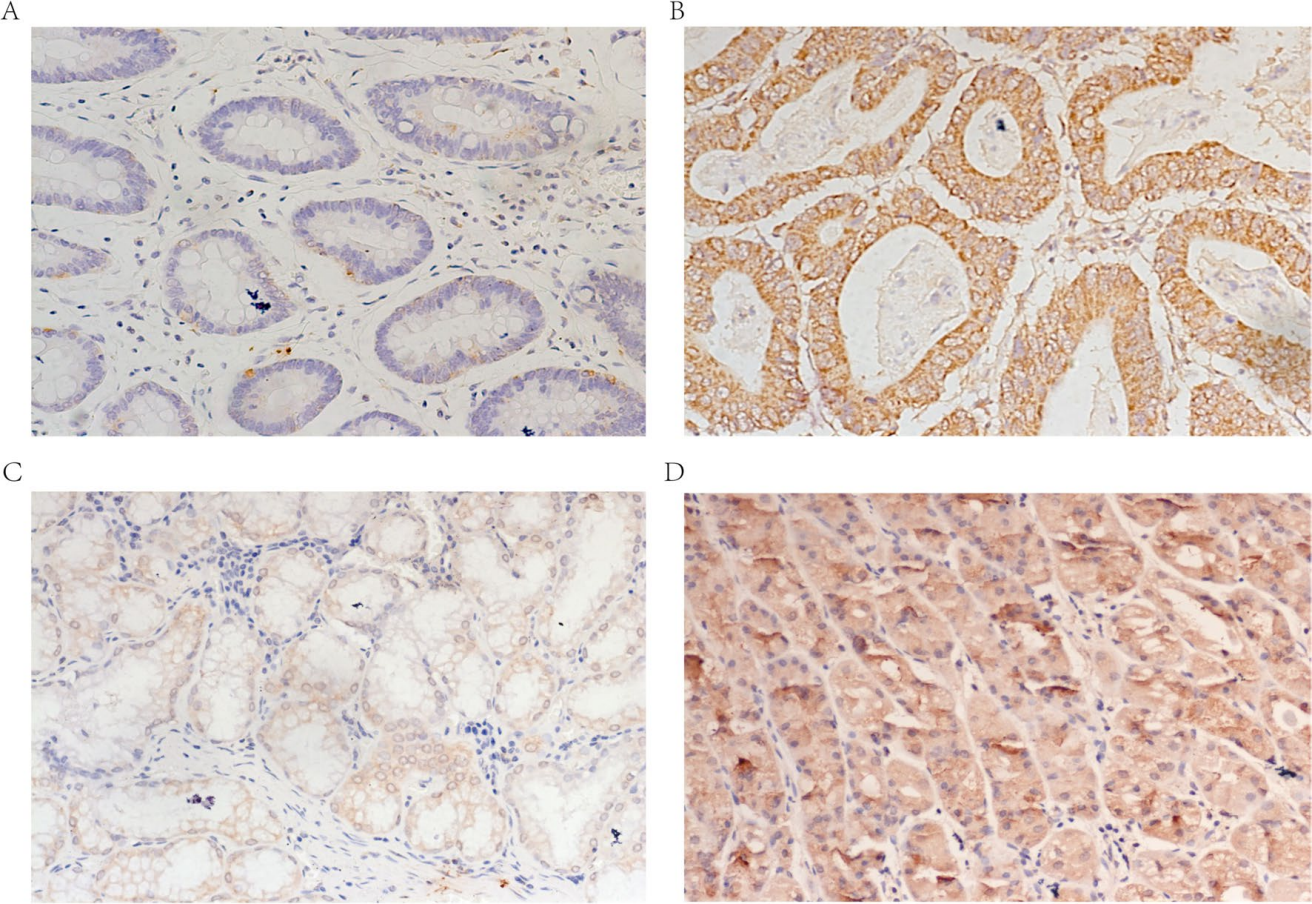
Representative immunohistochemical images of SERPINE 1 expression in gastric cancer (magnification: 200 x). SERPINE1 expression in gastric cancer tissues: **A** negative; **B** positive. SERPINE1 expression in normal tissues: **C** negative; **D** positive

To further verify the expression of SERPINE1 in gastric cancer tissues at the protein level, we performed experimental verification by immunohistochemistry. Through comparative analysis of the immunohistochemical results, we found that the expression of SERPINE1 in patients with GC was significantly higher than that in normal tissues (Fig. 3). SERPINE1-positive staining was mainly distributed in the cytoplasm of gastric cancer. We next analyzed the prognostic values of SERPINE1 expressions using the follow-up data of the 20 patients with GC. Kaplan–Meier analysis showed that patients with up-regulated SERPINE1 had significantly shorter survival (*P* < 0.05 Table 1). SERPINE1 expression in tumor tissues was positive in 12 cases and positive in the adjacent tissues in 5 cases. According to statistical analysis, SERPINE1 expression in tumor tissues and adjacent normal tissues was statistically different (*P* < 0.05).

**Table 1:** Correlations between SERPINE1 expression and clinicopathologic parameters in patients with GC

	Number of cases	Expression of SERPINE1		*p* value	*χ*^2^
		Low	High		
Age					
< 65	12	5	7		
≥ 65	8	3	5	> 0.05	0.612
Sex					
Male	17	6	11		
Female	3	2	1	> 0.05	0.344
T stage					
T1 + T2	4	2	2		
T3 + T4	16	6	10	> 0.05	0.535
Prognosis					
Survival	8	6	2		
Death	12	2	10	< 0.05	0.015

### SERPINE1 may inhibit the cuproptosis process SERPINE1

**Fig. 4:**
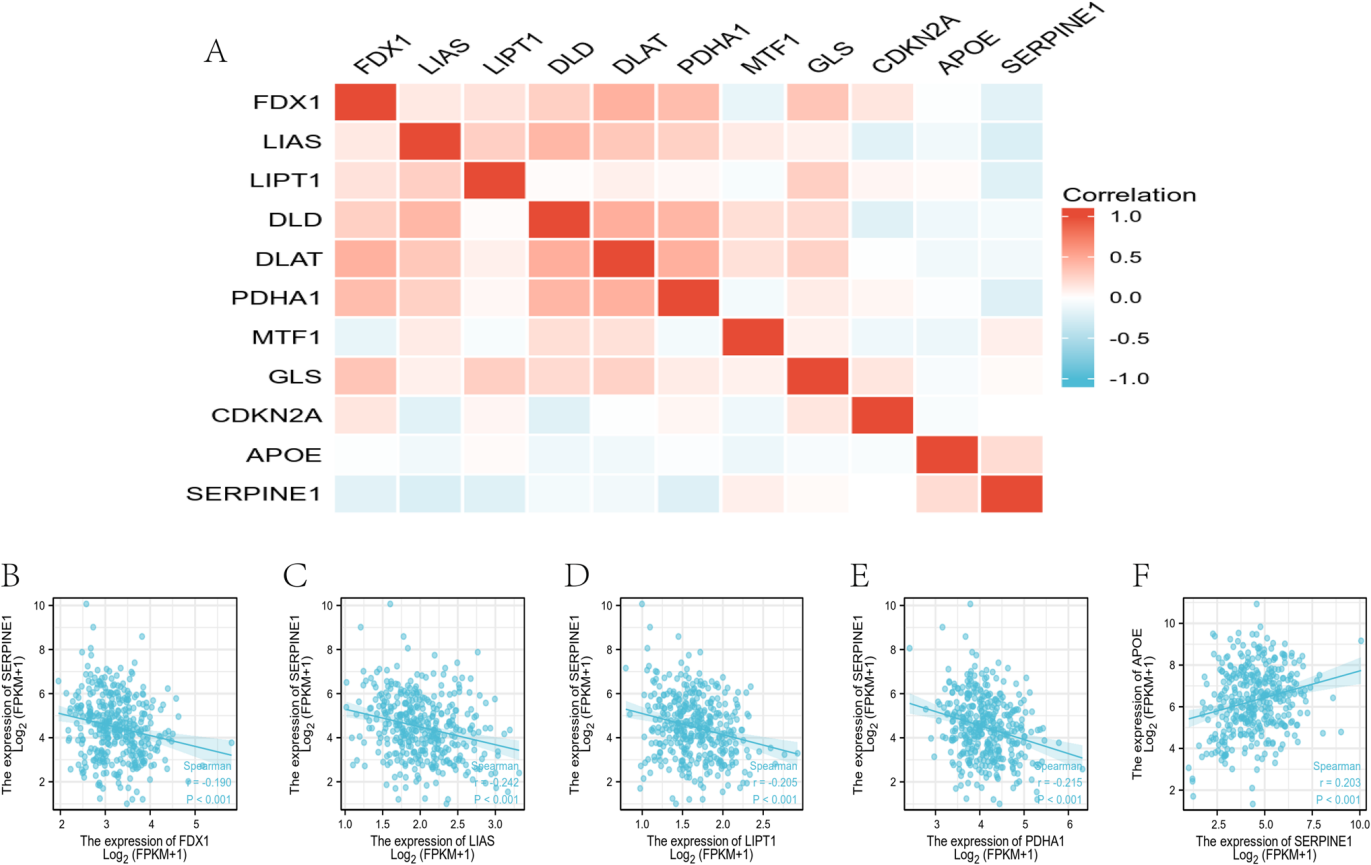
Correlation analysis of cuproptosis-related genes. **A** Analysis of the correlation between SERPINE1 and cuproptosis-related genes. **B**–**F** Correlation analysis of SERPINE1 with **B** FDX1, **C** LIAS, **D** LIPT1, **E** PDHA1, and **F** APOE

Cuproptosis is a novel identified regulated cell death, which is correlated with the development, treatment response, and prognosis of cancer. SERPINE1 may inhibit the cuproptosis process and affect tumor progression. Spearman correlation analysis showed that SERPINE1 was negatively correlated with cuproptosis-related genes FDX1, LIAS, LIPT1, and PDHA1. On the contrary, SERPINE1 is positively correlated with APOE (Fig. 4A). Scatter plots show the correlation between SERPINE1 and FDX1 (*r* = 0.19), LIAS (*r* = 0.242), LIPT1 (*r* = 0.205), PDHA1 (*r* = 0.215), and APOE (*r* = 0.243), respectively (Fig. 3B–F). This is consistent with previous studies that showed that APOE mediates poor outcomes in tumor patients. In pan-cancer analysis, as a key regulator in copper-induced cell death, the transcription and protein expression of FDX1 were significantly reduced in most cancer types.

### SERPINE1 may have inhibitory immune microenvironment effects

**Fig. 5:**
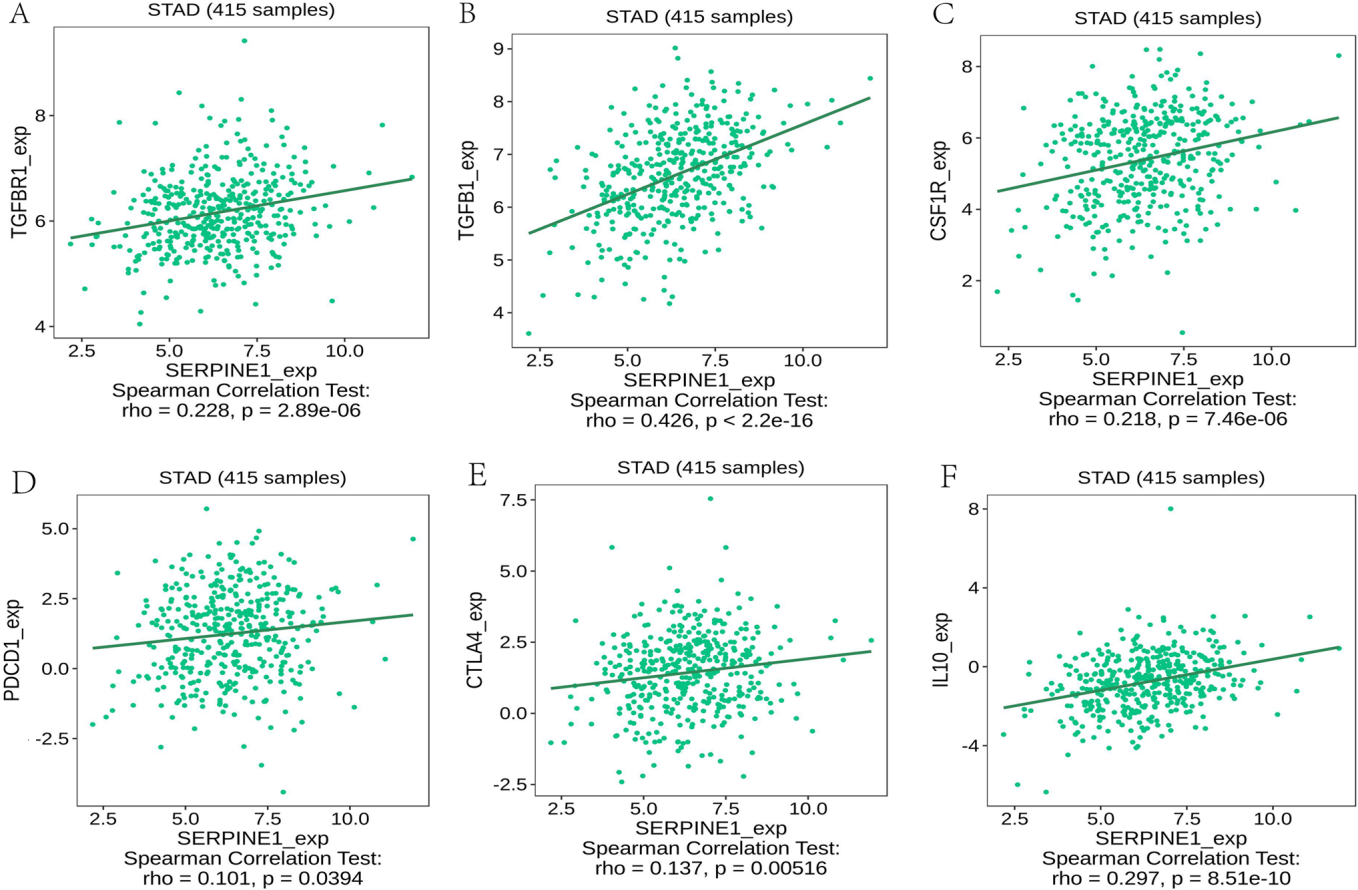
Association of SERPINE1 with immunoinhibitor in gastric cancer. **A** TGFBR1, **B** TGFB1, **C** CSF1R, **D** PDCD1, **E** CTLA4, and **F** IL-10

**Fig. 6:**
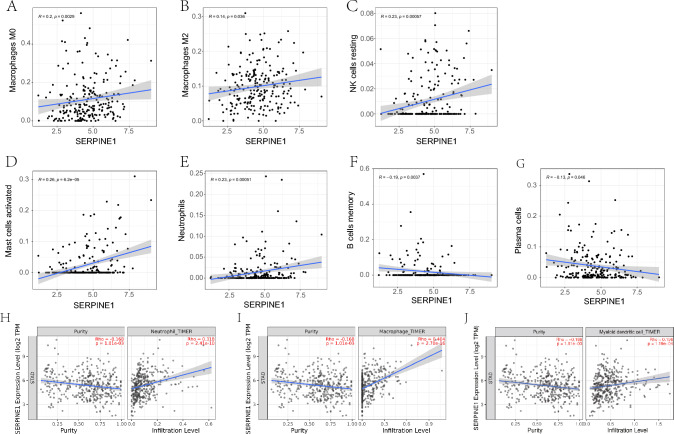
Correlation of SERPINE1 expression with immune infiltration level in gastric cancer. **A**–**F** Correlation between immune cell. **A** macrophages M0, **B** macrophages M2, **C** resting NK cells, **D** activated mast cells, **E** neutrophils, **F** B cells memory, and **G** plasma cells. **H–J** TIMER 2.0 SERPINE1 correlation with **H** neutrophil, **I** macrophages, and **J** myeloid dendritic

Previous studies have shown that the immune system is significantly related to the development of tumors. Therefore, we further explored whether SERPINE1 affects immune factors. After screening the cuproptosis-related gene SERPINE1, the role of SERPINE1 in the immune microenvironment was further explored. SERPINE1 expression is associated with immune infiltration. Immunosuppressants CSF1R, IL10, KDR, PDCD1LG2, TGFB1, and TGFBR1 were positively correlated with SERPINE1 expression in the TISIDB database (*P* < 0.05 Fig. 5). Based on the "CIBERSORT" analysis, the difference in immune cell infiltration level between the high and low SERPINE1 expression groups showed that there was a significant correlation between SERPINE1 and tumor-infiltrating immune cells. The infiltration level of resting NK cells, neutrophils, activated mast cells, and macrophages M2 was positively correlated with SERPINE1. However, B cells memory and plasma cells were negatively correlated with SERPINE1 (Fig. 6A–G). Further analysis of SERPINE1 expression by the TIMER algorithm showed that the invasion of neutrophils, macrophages, and myeloid dendritic was positively correlated with SERPINE1 (Fig. 6H–J). In addition, the TIMER database showed that this gene was differentially expressed in breast cancer, colon cancer, and renal clear cell carcinoma (Supplementary Fig. 1).

### SERPINE1 was enriched in P53, Pi3k/Akt, and TGF-β signaling pathway that affects cuproptosis and immunosuppression

**Fig. 7:**
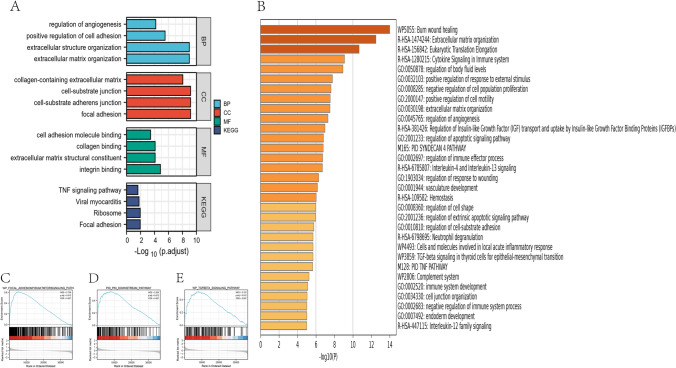
Functional enrichment analysis. **A** GO, KEGG functional enrichment analysis. **B** GO, KEGG functional enrichment analysis In Metascape database. **C**–**E** GSEA analysis showed that the up-regulated SERPINE1 was mainly concentrated in the **C** P53 pathways, **D** Pi3k/Akt pathways, **F** TGF-β pathways. GO, gene ontology; BP, biological process; CC, cellular component; MF, molecular function. NES, normalized enrichment fraction, concentration fraction; FDR, false discovery rate

To better understand the function of differentially expressed genes, GO and KEGG pathway enrichment analysis was performed. GO enrichment analysis showed that biological behavior was related to the positive regulation of the extracellular matrix, extracellular structural organization, and cell adhesion. Also, KEGG enrichment analysis showed that SERPINE1 was enriched in the TNF signaling pathway (Fig. 7A). SERPINE1 has been associated with the extracellular matrix, angiogenesis, and apoptosis regulation in the Metascape database. It was enriched in TNF, TGF-β, and IL-12 signaling pathways (Fig. 7B), and in the larger enrichment pathway, Pi3k/Akt and Wnt signaling pathways were included (Supplementary Fig. 2A).

To further explore the relationship between the enrichment pathway and the prognosis of GC patients, GSEA analysis showed that SERPINE1 was enriched in P53, Pi3k/Akt, TGF-β, and other pathways (Fig. 7C–E). Finally, multi-pathway analysis using the "GSVA" package in R software showed that SERPINE1 was positively correlated with angiogenesis, apoptosis, ECM degradation, inflammation, p53, and Pi3k–Akt pathways, and negatively correlated with the citric acid cycle (Supplement Fig. 2B–G).

### Drug sensitivity assessment

**Fig. 8:**
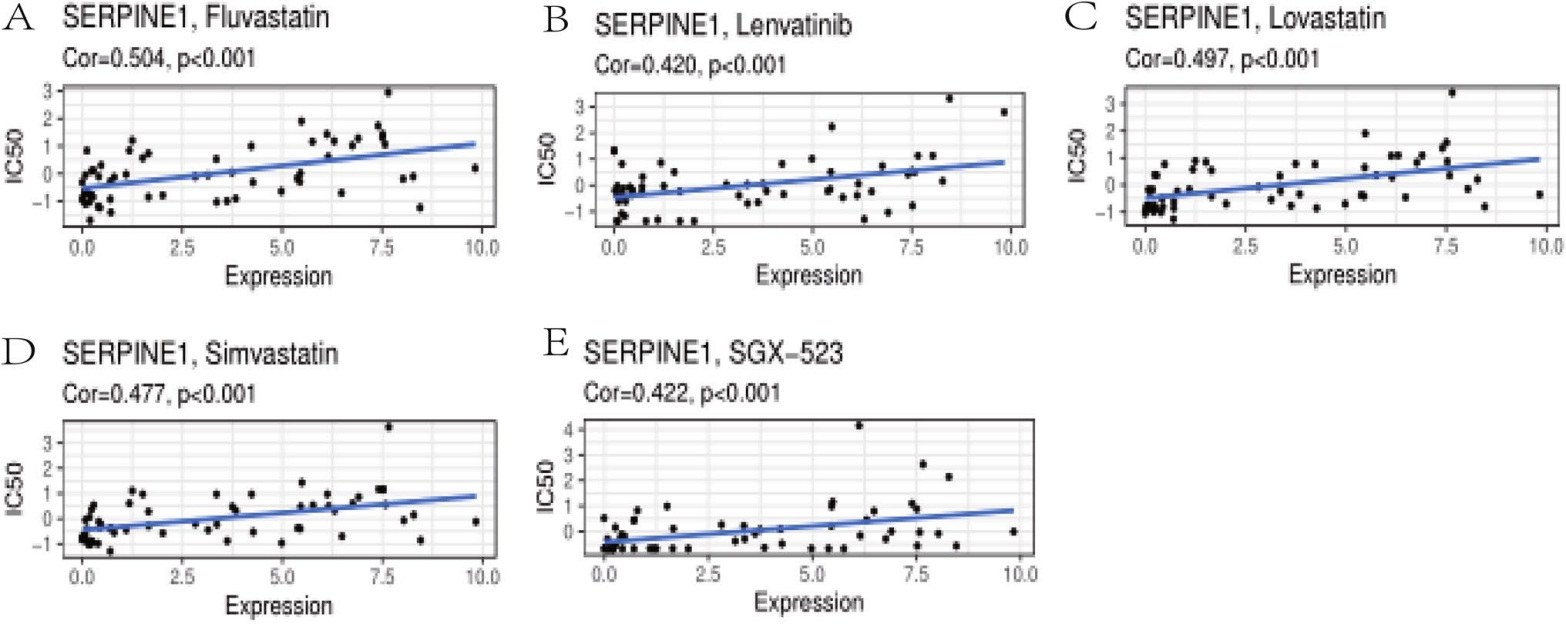
Drug sensitivity assessment. **A**–**E** Drug sensitivity assessment of **A** fluvastatin, **B** lenvatinib, **C** lovastatin, **D** simvastatin, and **E** SGX-523

After FDA screening, 75 of the drugs went through clinical trials and 188 were approved by the FDA. The gene expression of the finished NCI-60 cell line showed that 23,805 genes were expressed in 60 different tumor cell lines. The scatter plot shows that the drugs that are positively related to SERPINE1 are: Fluvastatin, Lovastatin, Simvastatin, SGX-523, and Lenvatinib (Fig. 8A–E). Given these findings, these small molecule compounds may be potential therapeutic agents for SERPINE1, but further analysis is needed shortly. Our results provide potential molecular chemotherapy compounds for patients with GC.

### Establishing a risk model and analysis of the prognostic value

**Fig. 9:**
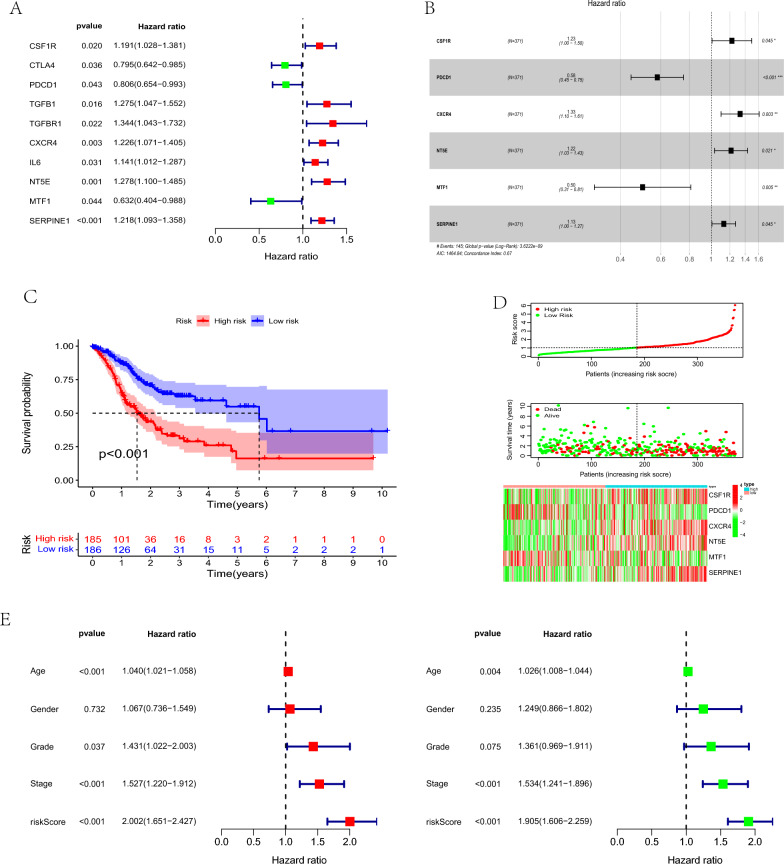
Establishment of prognostic risk profiles. **A** Univariate Cox regression analysis. **B** Multivariate Cox regression analysis. **C** Survival curves were drawn based on high-risk and low-risk groups. **D** Risk heat map and risk curve. **E** Univariate and multivariate Cox analyses of clinicopathologic characteristics (age, sex, stage, T, N, M), and risk characteristics in GC patients

We used univariate Cox regression analysis to screen out seven genes associated with immunity combined with cuproptosis in GC (*P* < 0.05). It included four potential risk genes (CSF1R, TGFB1, TGFBR1, SERPINE1) and three potential protection genes (CTLA4, PDCD1, MTF1; Fig. 9A). Multivariate Cox regression analysis was performed and two of them were identified as potential risk genes and two were identified as potential protective genes (Fig. 9B). Then the survival curve, risk curve, and risk heat map were drawn according to the high- and low-risk groups. The survival curve showed that the survival of the high- and low-risk groups was different (*P* < 0.05 Fig. 9C). The risk curve and risk heat map showed that with the increase in risk score, the prognosis of patients was worse (Fig. 9D-F).

To verify whether risk score and clinical features can be used as independent prognostic factors, we combined the risk score and clinical data to conduct univariate and multivariate independent prognostic analysis and determined that the factors that can be used as independent prognostic factors are age, stage, and risk score (both univariate and multivariate *P* < 0.05) (Fig. 9G).

**Fig. 10:**
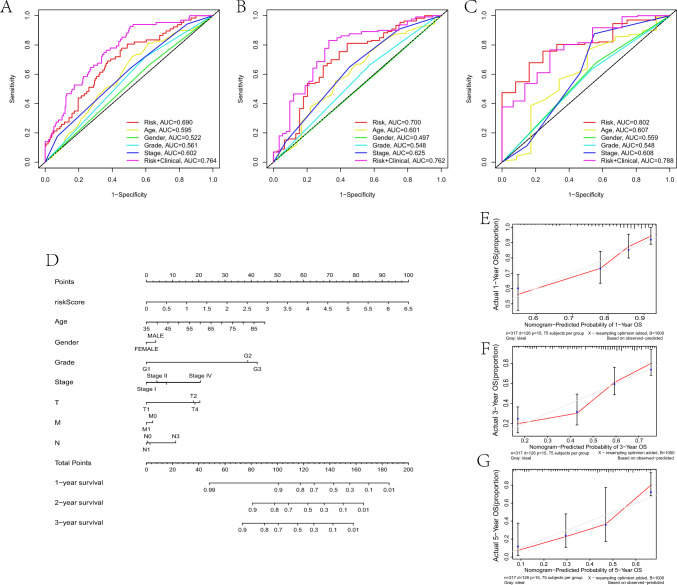
ROC curve, calibration curves, and nomogram. **A**–**C** The 1-, 3-, and 5-year area under the curve (AUC) of ROC curves. **D** A nomogram for prediction of the 1-, 3-, and 5-year overall survival rates of patients with gastric cancer. **E**–**G** Calibration curves of the nomogram prediction of the 1-, 3-, and 5-year overall survival rates of patients with gastric cancer. ROC, receiver operating characteristic; TPR, true positive rate; FPR, false positive rate; AUC, area under the curve

To provide clinicians with a better quantitative method to predict the prognosis of patients with GC, we developed a column graph combining age, sex, N, T, and risk score (Fig. 10A). The line graph shows that risk score is an important factor in various clinical parameters, and the constructed calibration curve shows that the line graph matches the actual survival rate of GC patients very well. On the nomogram, a higher total number of points was associated with a worse prognosis. Additionally, calibration curves were used to assess the prediction efficacy of the nomogram (Fig. 10B-D). Time-dependent ROC analysis showed that the prognostic accuracy of OS was 0.744 at 1 year, 0.756 at 3 years, and 0.774 at 5 years (Fig. 10E–G). As a result, it was clear that the model we established had acceptable accuracy in predicting the prognosis in the test set. As a result, the model established by us has excellent accuracy in predicting the prognosis.

### Validation of the prognostic model of the test set

To verify the accuracy of the established prognostic model, we obtained 183 gastric cancer patients from GEO (GSE15459) and calculated the risk score using the same formula as that in the training set. According to the median value of the risk score, 96 patients in the GEO cohort were classified into the low-risk group and 87 patients into the high-risk group. In addition, Kaplan–Meier analysis also indicated that the low-risk group and the high-risk group had significantly different survival rates (*P* < 0.05 Supplementary Fig. 3A), which was consistent with the result in the training set. As a result, it was clear that the model we established has acceptable accuracy in predicting prognosis in the test set.

## Discussion

Gastric cancer is one of the most common cancers, with a high mortality-to-incidence ratio. In recent years, the morbidity and mortality of young patients have been increasing year by year (Wong et al. 2021). Copper, as an important cofactor of several metalloenzymes that promote tumor metastasis, has recently become an emerging subject in the development of antitumor therapies. There is increasing evidence that unbalanced copper homeostasis can affect tumor growth and induce tumor cell death, and copper plays an indispensable role in tumor immunity and antitumor therapy. Generally speaking, the accumulation of copper will lead to the destruction of the copper balance and promote the occurrence of cuproptosis. However, the occurrence of copper accumulation in cancer does not trigger the death of copper cells, but activates the pro-cell proliferation pathway and participates in driving the pathological features of cancer. Previous studies have shown that the immune microenvironment plays a crucial role in the progression of cancer. Cell death releases inflammatory factors, and tumor cells immune escape, providing a suitable living environment for tumor cells (Tiwari et al. 2022). At present, in addition to the classical cuproptosis pathway-mediated genes, a large number of cuproptosis-related genes also have cuproptosis-related characteristics and participate in the formation of the immune microenvironment, mediating the poor prognosis of tumor patients. SERPINE1 acts as a tumor suppressor in colon cancer cells through immunosuppression (Wang et al. 2021). However, it is not clear how SERPINE1 mediates tumor progression in GC. Here, we describe the role of the cuproptosis-related gene SERPINE1 in GC and its immune microenvironment and how it may mediate pathways that influence tumor progression.

SERPINE1 is a member of the serine protease inhibitor family. It is the primary inhibitor of tissue plasminogen activator and urokinase-plasminogen activator. Studies have shown that high SERPINE1 expression is associated with malignant biological behavior in tumors. This experiment also verifies that the expression of SERPINE1 in patients with GC was significantly higher than that in normal tissues. However, SERPINE1 was not associated with factors such as age and gender. Patients with high SERPINE1 expression had a poor prognosis. SERPINE1 was an independent prognostic indicator. Our study was consistent with the previous studies, and analysis of the public databases (TCGA and GEO) supported our conclusions. Therefore, SERPINE1 can be used as a new biomarker and therapeutic target for GC, providing a new therapeutic direction for the treatment of GC.

SERPINE1 has been implicated in the regulation of fibrinolysis pathways, cardiovascular disease, inflammation, thrombosis, and cancer progression. SERPINE1 itself has metal–ligand sites. Cu(II) can coordinate with a high-affinity binding site containing two residues near the N terminus of SERPINE1 (Bucci et al. 2016). Previous work has shown that two residues near the N terminus, H2 and H3, are located at a high-affinity copper-binding site in SERPINE1. In current further studies, adjacent residues H10, E81, and H364 have also been tested as possible sites involved in the coordination of the high-affinity site Cu(II) (Chu et al. 2021). According to the gene correlation analysis, SERPINE1 is negatively correlated with cuproptosis-related genes such as FDX1 and LIPT1. FDX1 is a key regulator of cell death induced by copper cells, and FDX1 is the upstream regulator of protein sulfenylation modification. On the one hand, FDX1 is involved in the regulation of protein sulfenylation. FDX1, on the other hand, reduces Cu^2+^ to the more toxic Cu^+^, inducing cell death. The low expression of FDX1 in most tumors and the up-regulation of FDX1 in liver cancer (Zhang et al. 2022), colon cancer (Wang et al. 2022), kidney cancer (Huang et al. 2022), and other bioinformatics analysis can mediate the good prognosis of tumor patients. Similarly, LIPT1 is a key enzyme in the lipoic acid pathway. LIPT1 expression was increased in melanoma biopsies and was an independent favorable prognostic indicator for melanoma patients (Lv et al. 2022). In contrast, SERPINE1 is positively correlated with APOE. Similar to the poor prognosis in patients mediated by APOE (Huang et al. 2022), SERPINE1 and APOE are biomarkers of bladder cancer, both of which are highly expressed and mediate poor prognosis in patients (Zhang et al. 2014).

According to previous studies, SERPINE1 is associated with immune cell infiltration and plays a role in colon cancer microenvironment remodeling and immune cell infiltration (Wang et al. 2021). Interestingly, we have shown through TISIDB and further TIMER analysis that SERPINE1 expression is positively correlated with immune infiltration and influences immune regulation. SERPINE1 is associated with several microenvironmental factors that mediate the maintenance of inhibitory immunity, such as TGFBR1, TGFB1, CSF1R, and IL-10. TGFB1 belongs to the TGF-β family and is a common immunosuppressive medium that promotes tumor progression. The immunosuppressive effect of the TGF-β signaling pathway promotes tumor progression. The development of TGF-β inhibitors as antitumor drugs has been studied in the context of breast cancer, colon cancer, esophageal cancer, and other cancers (Wrzesinski et al. 2007). SERPINE1 has been described as a classic marker of TGF-β pathway activation in glioblastoma, and up-regulation of SERPINE1 has been associated with lower survival rates and poor clinical prognosis (Hau et al. 2011). Similarly, the inhibitory immune microenvironment within the tumor shows up-regulation of the IL-10 factor, which is involved in inflammation and immunosuppression and promotes immune evasion. In further validation of the TIMER database, macrophage cell invasion and neutrophil invasion were positively correlated with SERPINE1. Previous studies have shown that the increase of SERPINE1 may mediate the transformation of pro-inflammatory M1 macrophages into anti-inflammatory M2 macrophages through the p38MAPK/NF‐κB/IL-6 pathway (Baumeier et al. 2021). M2 phenotype macrophages play an immunoregulatory role, such as participating in inducing Th2 response, promoting tumor angiogenesis, and accelerating local lymph node metastasis. This study is consistent with previous studies, showing the inhibitory immune microenvironment induction effect of SERPINE1 in GC tissues.

To analyze how SERPINE1 affects the progression of GC patients, we conducted a functional analysis to explore the potential biological mechanism. GO results showed that SERPINE1 is enriched in the extracellular matrix and related to angiogenesis and apoptosis regulation. This further validates that SERPINE1 could maintain proliferation signals in tumors, promote tumor cell migration, and inhibit tumor cell apoptosis. KEGG enrichment analysis and GSEA enrichment analysis showed that it was mainly enriched in the P53, Pi3k/Akt, and TNF pathways. Studies have found that these pathways are related to the regulation of apoptosis, immune response, and so on. In triple-negative breast cancer, the copper complex Cu(SBCM)2 may lead to cell cycle arrest and apoptosis through the p53 pathway (Foo et al. 2019). In the study of renal fibrosis, SERPINE1 also appears to up-regulate renal p53, since p53 protein levels are increased by sustained renal SERPINE1 expression and p53 stable suppression in SERPINE1 transductants, attenuating the induction of fibrotic factors, reversing the proliferative defects, and reducing the susceptibility to cell death. Sustained SERPINE1 expression contributes to epithelial dedifferentiation, G2/M proliferative arrest, fibrogenesis, and apoptosis (Gifford et al. 2021). In addition, P53, as an important immune-related pathway, is involved in the formation of tumor immune microenvironment. The deletion or mutation of p53 in cancer can affect the recruitment and activity of bone marrow cells and T cells, thus allowing immune escape and promoting cancer progression. p53 can also play a role in immune cells, producing a variety of outcomes that may impede or support tumor development. Disulfiram has copper‐dependent anticancer properties in vitro and in vivo. DSF/Cu inhibited both NF‐κB and TGF‐β signaling, including the nuclear translocation of NF‐κB subunits and the expression of Smad4, leading to the down‐regulation of Snail and Slug, which contributed to phenotype EMT. These results indicated that DSF/Cu suppressed the metastasis and EMT of hepatic carcinoma through TGF‐β signaling (Li et al. 2018).

TGF-β cytokines are crucial mediators of immune homeostasis that inhibit expansion and functions of different immune cell types such as effector T cells, macrophages, natural killer (NK) cells, and antigen-presenting dendritic cells (DCs) (Wrzesinski et al. 2007; Batlle and Massagué 2019). Particularly, in glioblastoma, elevated TGF-β levels have often been associated with the immunosuppressed status of patients. TGF-β level reflects tumor immune surveillance ability (Hau et al. 2011). Activation of the tumor necrosis factor receptor-1 (TNF-R1) signaling pathway is involved in Cu-induced apoptosis and is characterized by significantly elevated mRNA and protein expression levels of the TNF-R1, FAS-associated death domain, TNFR-associated death domain, and cleaved caspase-8. The Pi3k/Akt signaling pathway can induce apoptosis of NSCLC cells and promote G0/G1 phase cell cycle arrest. Similarly, in triple-negative breast cancer, the up-regulation of SERPINE1 can induce the expression of lncRNA SOX2-OT, ultimately activating the Pi3k/Akt signaling pathway, which induces the migration and metastasis of triple-negative breast cancer cells (Zhang et al. 2022).

To further promote the prognostic value, we constructed a risk model and verified it in a validation cohort. We can see that in the final constructed model, PDCD1, MTF1, and SERPINE1 are cuproptosis-related genes, and CSF1R, PDCD1, and SERPINE1 are immune-related genes. Influencing tumor progression is a multi-factor complex process. The genes used in this study to establish risk profiles are strongly associated with tumor development and progression. Survival analysis indicated that the established risk profiles showed effective predictive performance for GC survival in both the training and validation cohorts. The ROC curve showed the reliability and stability of the risk curve. Finally, a column graph integrating risk scores and clinical features was also developed and calibrated and showed considerable predictive accuracy.

In addition, several potential small molecule compounds, SGX − 523, and statins (lovastatin, simvastatin, fluvastatin) were also screened for SERPINE1. The main function of statins is to inhibit HMG-CoA reductase and lower cholesterol. Cholesterol-lowering statins have known anticancer effects, for example, lovastatin can induce tumor cell apoptosis and inhibit tumor cell invasion and may cause cells to be stagnated in the G1 phase of the cell cycle. Statins can affect glioblastoma invasion and metastasis and induce cell apoptosis by inhibiting TGF-β activity (Xiao et al. 2019). In cancer cells, TGF-β drives malignant behavior, which stimulates EMT and promotes invasion, metastasis, and possible therapeutic resistance. Local production of TGF-β strongly inhibits antitumor immunity, and regulatory T cells inhibit the cytotoxicity of tumor-specific CD8 T cells through TGF-β signaling in vivo (Chen et al. 2005). SERPINE1 is a TGF-β target gene (Yamada-Nomoto et al. 2017). Therefore, statins may affect SERPINE1 expression through the TGF-β pathway. MET receptor tyrosine kinase inhibitor SGX523 specifically binds the c-Met protein, or hepatocyte growth factor receptor, blocks hepatocyte growth factor binding, and disrupts the MET signaling pathway. The drug induces the death of tumor cells expressing c-Met. c-Met is a receptor tyrosine kinase that is overexpressed or mutated in many tumor cell types and plays an important role in tumor cell proliferation, survival, invasion and metastasis, and tumor angiogenesis. SGX523 treatment inhibits c-MET-dependent brain tumor cell proliferation and G1/S cell cycle progression, and SGX523 also inhibits brain tumor cell migration and invasion (Guessous et al. 2010). These results suggest that these potential agents may provide new insights into the treatment of GC patients with high SERPINE1 expression.

At present, our study still has some limitations, such as a small clinical sample size, certain errors in the process of data collection, the direct mechanism of action of SERPINE1 in GC may not be detailed enough, and the molecular mechanism needs to be further verified by experiments and discussed in the follow-up study improvement.

## Conclusions

Cuproptosis-immune-related gene SERPINE1 was up-regulated in GC and closely related to the low overall survival rate. SERPINE1 expression was related to GC tumor immune microenvironment.

## Supplementary Information

Below is the link to the electronic supplementary material.

Supplementary file1 (PDF 40345 KB)

Supplementary file2 (PDF 1738 KB)

Supplementary file3 (PDF 9 KB)

## Data Availability

All data and R script in this study are available from the corresponding author upon reasonable request. All authors read and approved the final manuscript. Publicly available datasets were analyzed in this study, and these can be found in The Cancer Genome Atlas (https://portal.gdc.cancer.gov/) and Gene Expression (GSE13911, GSE15459), Genecard (https://www.genecards.org/), innateDB (https://www.innatedb.ca/).
