# Supplementary figures and images for: Cuproptosis-related gene SERPINE1 is a prognostic biomarker and correlated with immune infiltrates in gastric cancer
Source: J Cancer Res Clin Oncol. 2023 Jun 15;149(12):10851–65. doi: 10.1007/s00432-023-04900-1 (PMC10423162; doi:10.1007/s00432-023-04900-1)

SERPINE1 Expression Level (log2 TPM)

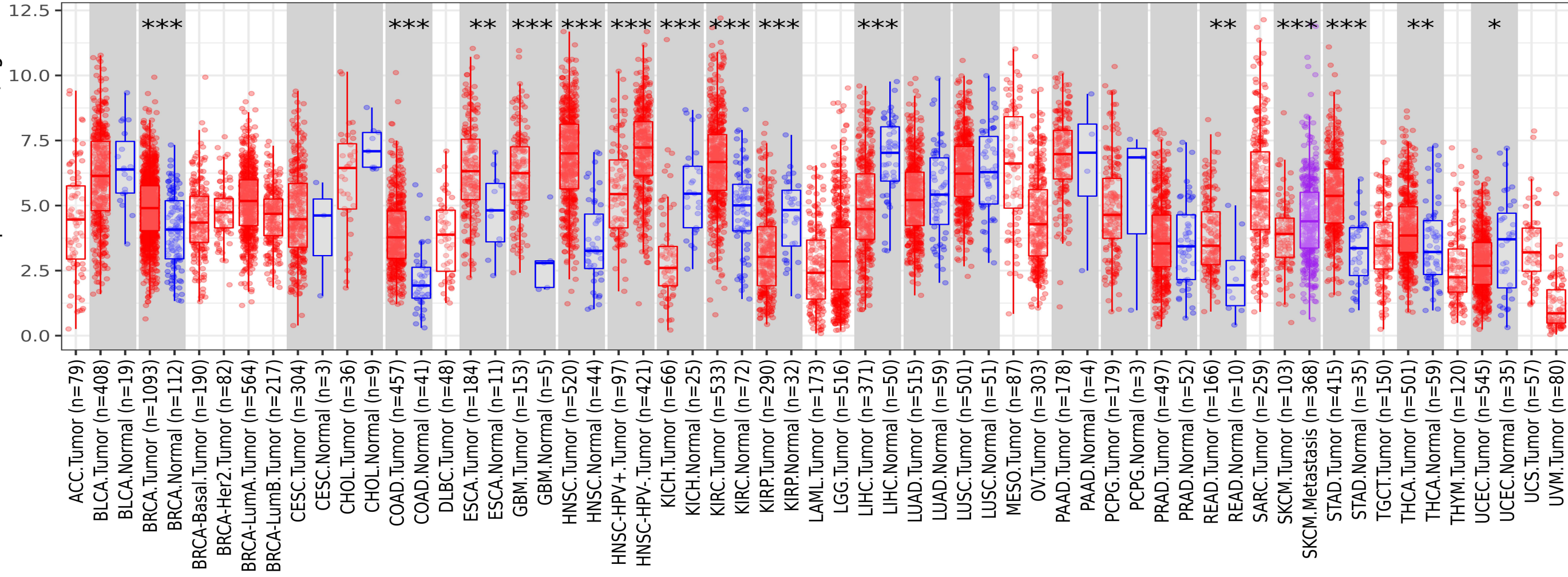

Supplement: Supplementary file 1 — Supplementary file1 (PDF 40345 KB) [file 432_2023_4900_MOESM1_ESM.pdf]

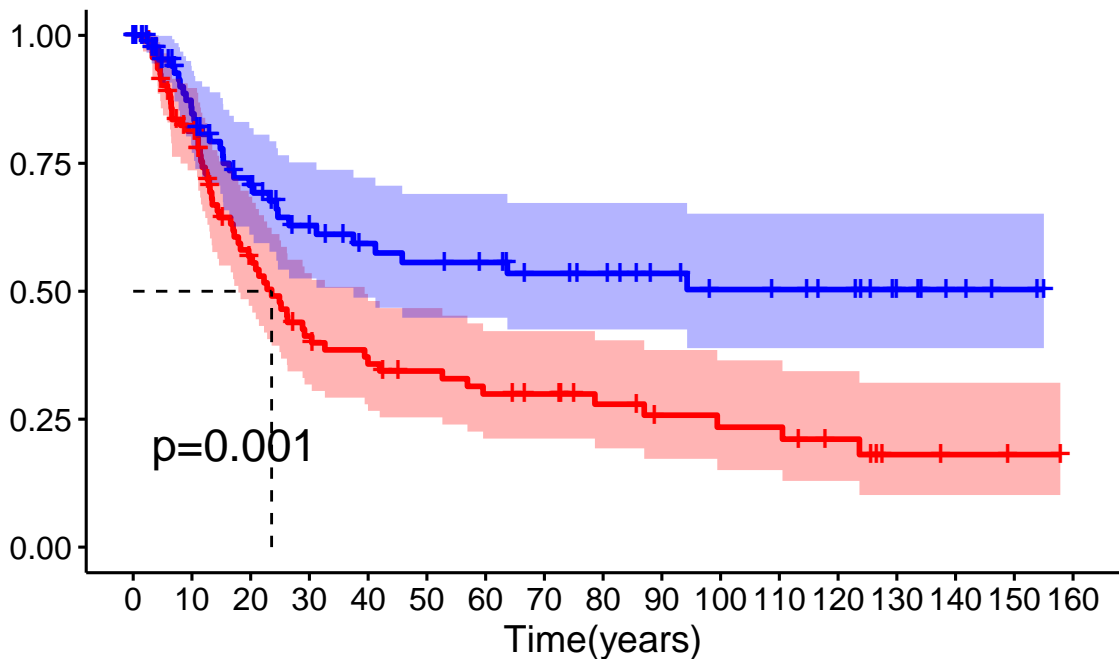

| Time (years) | High risk | Low risk |
|--------------|-----------|----------|
| 0            | 96        | 96       |
| 10           | 71        | 64       |
| 20           | 44        | 49       |
| 30           | 31        | 38       |
| 40           | 26        | 32       |
| 50           | 23        | 30       |
| 60           | 20        | 28       |
| 70           | 18        | 24       |
| 80           | 14        | 22       |
| 90           | 11        | 18       |
| 100          | 10        | 15       |
| 110          | 10        | 14       |
| 120          | 7         | 12       |
| 130          | 3         | 7        |
| 140          | 2         | 4        |
| 150          | 1         | 2        |
| 160          | 0         | 0        |

Supplement: Supplementary file 3 — Supplementary file3 (PDF 9 KB) [file 432_2023_4900_MOESM3_ESM.pdf]
