# Supplementary material for: Cuproptosis-related gene SERPINE1 is a prognostic biomarker and correlated with immune infiltrates in gastric cancer
Source: J Cancer Res Clin Oncol. 2023 Jun 15;149(12):10851–65. doi: 10.1007/s00432-023-04900-1 (PMC10423162; doi:10.1007/s00432-023-04900-1)
Supplement: Supplementary file 2 — Supplementary file2 (PDF 1738 KB) [file 432_2023_4900_MOESM2_ESM.pdf]

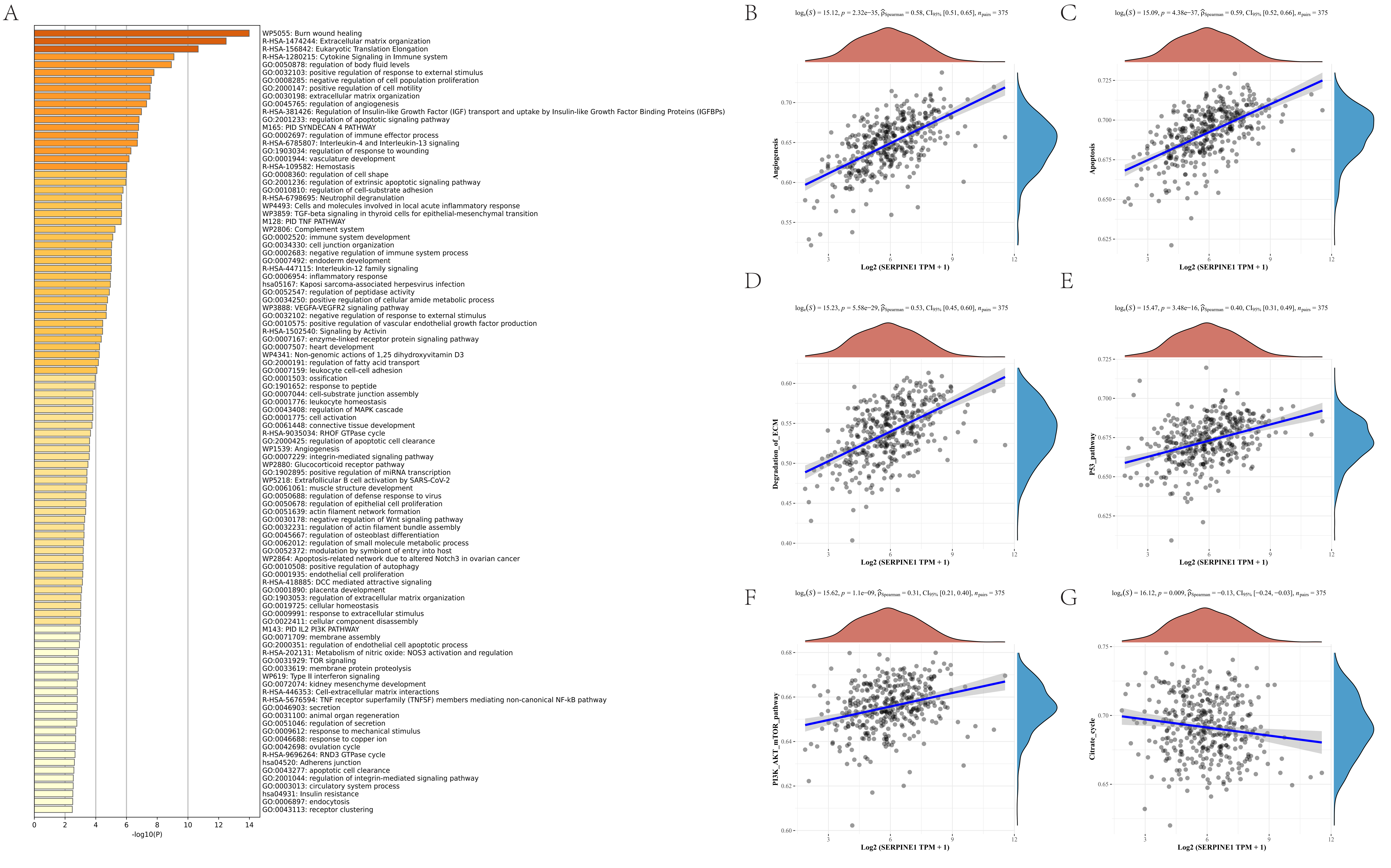
A
WP5055: Burn wound healing
R-HSA-1474244: Extracellular matrix organization
R-HSA-156842: Eukaryotic Translation Elongation
R-HSA-1280215: Cytokine Signaling in Immune system
GO:0050878: regulation of body fluid levels
GO:0032103: positive regulation of response to external stimulus
GO:0008285: negative regulation of cell population proliferation
GO:2000147: positive regulation of cell motility
GO:0030198: extracellular matrix organization
GO:0045765: regulation of angiogenesis
R-HSA-381426: Regulation of Insulin-like Growth Factor (IGF) transport and uptake by Insulin-like Growth Factor Binding Proteins (IGFBPs)
GO:2001233: regulation of apoptotic signaling pathway
M165: PID SYNDECAN 4 PATHWAY
GO:0002697: regulation of immune effector process
R-HSA-6785807: Interleukin-4 and Interleukin-13 signaling
GO:1903034: regulation of response to wounding
GO:0001944: vasculature development
R-HSA-109582: Hemostasis
GO:0008360: regulation of cell shape
GO:2001236: regulation of extrinsic apoptotic signaling pathway
GO:0010810: regulation of cell-substrate adhesion
R-HSA-6798695: Neutrophil degranulation
WP4493: Cells and molecules involved in local acute inflammatory response
WP3859: TGF-beta signaling in thyroid cells for epithelial-mesenchymal transition
M128: PID TNF PATHWAY
WP2806: Complement system
GO:0002520: immune system development
GO:0034330: cell junction organization
GO:0002683: negative regulation of immune system process
GO:0007492: endoderm development
R-HSA-447115: Interleukin-12 family signaling
GO:0006954: inflammatory response
hsa05167: Kaposi sarcoma-associated herpesvirus infection
GO:0052547: regulation of peptidase activity
GO:0034250: positive regulation of cellular amide metabolic process
WP3888: VEGFA-VEGFR2 signaling pathway
GO:0032102: negative regulation of response to external stimulus
GO:0010575: positive regulation of vascular endothelial growth factor production
R-HSA-1502540: Signaling by Activin
GO:0007167: enzyme-linked receptor protein signaling pathway
GO:0007507: heart development
WP4341: Non-genomic actions of 1,25 dihydroxyvitamin D3
GO:2000191: regulation of fatty acid transport
GO:0007159: leukocyte cell-cell adhesion
GO:0001503: ossification
GO:1901652: response to peptide
GO:0007044: cell-substrate junction assembly
GO:0001776: leukocyte homeostasis
GO:0043408: regulation of MAPK cascade
GO:0001775: cell activation
GO:0061448: connective tissue development
R-HSA-9035034: RHOF GTPase cycle
GO:2000425: regulation of apoptotic cell clearance
WP1539: Angiogenesis
GO:0007229: integrin-mediated signaling pathway
WP2880: Glucocorticoid receptor pathway
GO:1902895: positive regulation of miRNA transcription
WP5218: Extrafollicular B cell activation by SARS-CoV-2
GO:0061061: muscle structure development
GO:0050688: regulation of defense response to virus
GO:0050678: regulation of epithelial cell proliferation
GO:0051639: actin filament network formation
GO:0030178: negative regulation of Wnt signaling pathway
GO:0032231: regulation of actin filament bundle assembly
GO:0045667: regulation of osteoblast differentiation
GO:0062012: regulation of small molecule metabolic process
GO:0052372: modulation by symbiont of entry into host
WP2864: Apoptosis-related network due to altered Notch3 in ovarian cancer
GO:0010508: positive regulation of autophagy
GO:0001935: endothelial cell proliferation
R-HSA-418885: DCC mediated attractive signaling
GO:0001890: placenta development
GO:1903053: regulation of extracellular matrix organization
GO:0019725: cellular homeostasis
GO:0009991: response to extracellular stimulus
GO:0022411: cellular component disassembly
M143: PID IL2 PI3K PATHWAY
GO:0071709: membrane assembly
GO:2000351: regulation of endothelial cell apoptotic process
R-HSA-202131: Metabolism of nitric oxide: NOS3 activation and regulation
GO:0031929: TOR signaling
GO:0033619: membrane protein proteolysis
WP619: Type II interferon signaling
GO:0072074: kidney mesenchyme development
R-HSA-446353: Cell-extracellular matrix interactions
R-HSA-5676594: TNF receptor superfamily (TNFSF) members mediating non-canonical NF-kB pathway
GO:0046903: secretion
GO:0031100: animal organ regeneration
GO:0051046: regulation of secretion
GO:0009612: response to mechanical stimulus
GO:0046688: response to copper ion
GO:0042698: ovulation cycle
R-HSA-9696264: RND3 GTPase cycle
hsa04520: Adherens junction
GO:0043277: apoptotic cell clearance
GO:2001044: regulation of integrin-mediated signaling pathway
GO:0003013: circulatory system process
hsa04931: Insulin resistance
GO:0006897: endocytosis
GO:0043113: receptor clustering
0 2 4 6 8 10 12 14
-log10(P)
B
loge(S) = 15.12, p = 2.32e−35, ρ̂Spearman = 0.58, CI95% [0.51, 0.65], npairs = 375
Angiogenesis
Log2 (SERPINE1 TPM + 1)
C
loge(S) = 15.09, p = 4.38e−37, ρ̂Spearman = 0.59, CI95% [0.52, 0.66], npairs = 375
Apoptosis
Log2 (SERPINE1 TPM + 1)
D
loge(S) = 15.23, p = 5.58e−29, ρ̂Spearman = 0.53, CI95% [0.45, 0.60], npairs = 375
Degradation_of_ECM
Log2 (SERPINE1 TPM + 1)
E
loge(S) = 15.47, p = 3.48e−16, ρ̂Spearman = 0.40, CI95% [0.31, 0.49], npairs = 375
P53_pathway
Log2 (SERPINE1 TPM + 1)
F
loge(S) = 15.62, p = 1.1e−09, ρ̂Spearman = 0.31, CI95% [0.21, 0.40], npairs = 375
PI3K_AKT_mTOR_pathway
Log2 (SERPINE1 TPM + 1)
G
loge(S) = 16.12, p = 0.009, ρ̂Spearman = −0.13, CI95% [−0.24, −0.03], npairs = 375
Citrate_cycle
Log2 (SERPINE1 TPM + 1)
